# Tree ring evidence of rapid development of drunken forest induced by permafrost warming

**DOI:** 10.1111/gcb.16176

**Published:** 2022-04-07

**Authors:** Kazumichi Fujii, Koh Yasue, Yojiro Matsuura

**Affiliations:** ^1^ 57880 Forestry and Forest Products Research Institute Tsukuba Japan; ^2^ Institute of Mountain Science Shinshu University Nagano Japan

**Keywords:** climate warming, cryoturbation; dendrochronology, gelisols, lignin, permafrost, thermokarst

## Abstract

Black spruce trees growing on warming permafrost lean in all directions due to soil movement, forming a “drunken” forest. Two hypothetical drivers of drunken forest development are (i) loosening of the soil foundation induced by permafrost degradation in warm summers and (ii) mound rising induced by freezing soil in winter. However, no evidence has previously clarified whether recent tree leaning is related to climate warming or is part of a natural hummock formation process. Here, we provide evidence that tree leaning and soil hummock formation have accelerated due to climate warming. We find that trees’ leaning events synchronize with the development of soil hummocks as recorded in tree rings with lignin‐rich cells. Tree leaning is caused by mound rising in winter due to refreezing of soil following deep thaws in summer, rather than by loosening of the soil foundation in summer. Hummock formation shifted from periodic events before 1960 to continuous mound rising in the warmer succeeding 50 years. Although soil change is generally a slow process, recent permafrost warming has induced rapid hummock formation, which threatens the stability of drunken forests and organic carbon in soil hummocks based on shallow permafrost table.

## Graphical abstract

We found that tree leaning and soil hummock formation have accelerated due to recent climate warming. Development of the drunken forest is caused by the growth of soil hummocks before 1960, but hummock formation shifted from periodic events to continuous mound rising in the warmer succeeding 50 years. Recent warming threatens the stability of drunken forests and organic carbon stored in soil hummocks even in the continuous permafrost area.

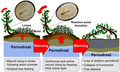

## 1 INTRODUCTION

**FIGURE 1:**
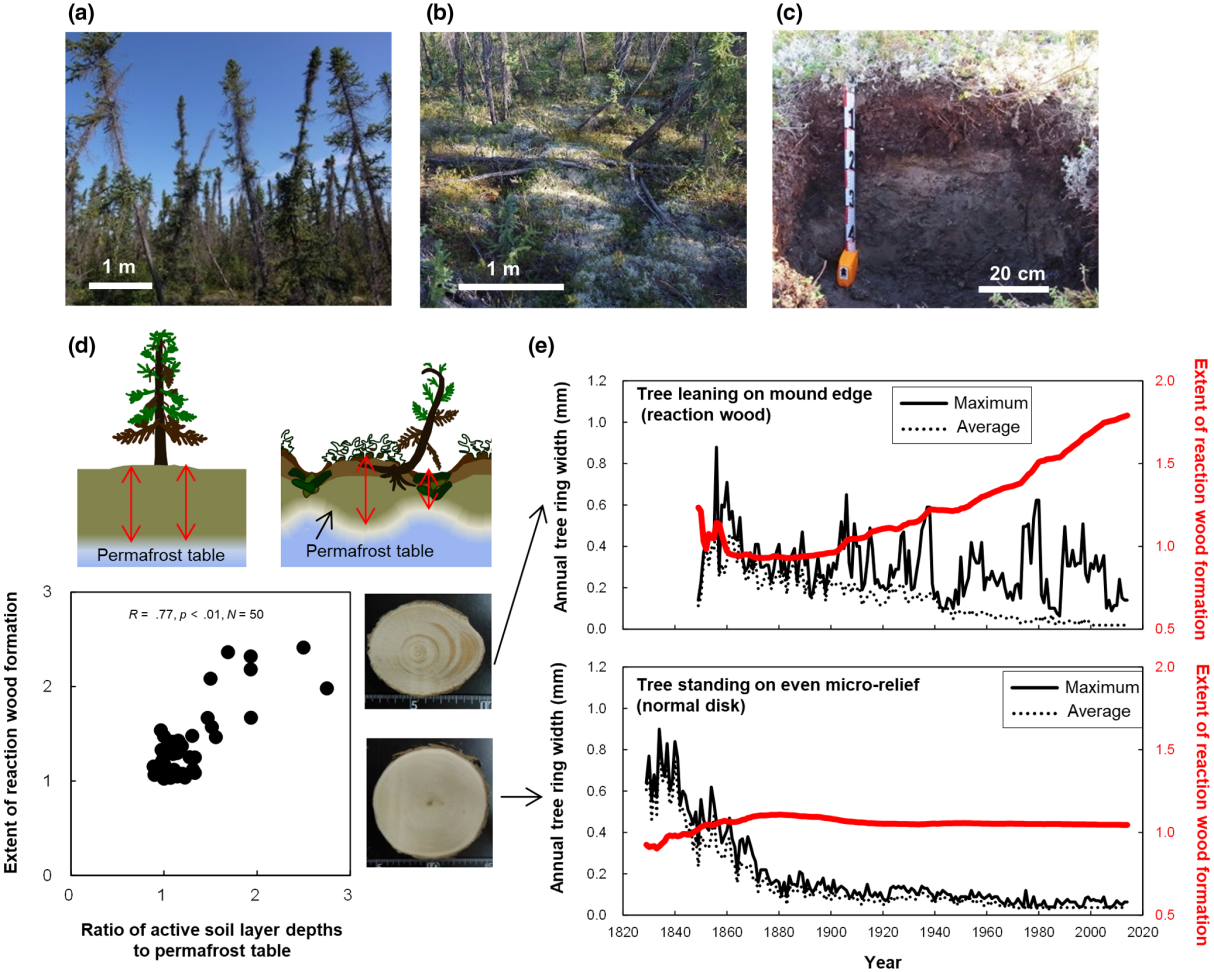
Drunken forest and soil hummocks on permafrost layer. (a) Drunken forest. (b) Hummocky micro‐relief. (c) Soil mound profile on permafrost layer. (d) Relationship between magnitude of tree leaning (ratios of maximum to average wood radius) and mound size indicator (ratios of active layer depths to permafrost table). (e) Maximum and average tree ring widths and extent of reaction wood formation

Circumpolar permafrost ecosystems have been increasingly exposed to climate warming in recent decades (Post et al., 2013). With the visible loss of arctic ice, the unseen loss of permafrost has also been predicted by several global models (Koven et al., 2011; Post et al., 2013; Torre Jorgenson et al., 2013). Trees growing on warming permafrost tend to lean in all directions due to soil movement, forming “drunken” forests, which have been observed in several regions (Figure 1a). Thus, drunken forests are widely regarded as a symptom of permafrost degradation (Schuur & Abbott, 2011); however, the effects of climate warming on permafrost‐affected soil and drunken forest development remain to be clarified in remote areas with limited data availability.

In northern permafrost areas, drunken black spruce forests tend to grow in thin active layers underlain by a hummocky microrelief of permafrost‐affected soil (Figure 1b,c; Zoltai, 1975). These conditions require soil movement driven by ice freeze–thaw cycles and heaving of the shallow permafrost table (Kokelj & Burn, 2004; Kokelj et al., 2007). The development of polygonal soil mounds (diameter, 1–2 m) is caused mainly by ice wedge formation during repeated freeze–thaw cycles at a geological time scale (i.e., thousands of years; Walker et al., 2008) but is also promoted biologically by the accumulation of lichen, moss debris, and humus in forest fire regeneration cycles (i.e., several hundreds of years) on the shallow permafrost table (Daanen et al., 2012; Fujii et al., 2020; Ping et al., 2008). Therefore, soil movement driven by ice and biological activity is highly dependent on climate.

Two hypotheses of tree leaning are (1) a loosening of the soil foundation is induced by permafrost melting in warming summer and (2) the rise of mounds is caused by freezing soil in winter. Regarding the first hypothesis, permafrost degradation caused by warming can increase the leaning of black spruce trees as permafrost thawing destabilizes the surface soil on which the trees stand. The second hypothesis is based on natural hummock formation caused by freeze–thaw cycles or upward pressure generated by frost heaving in winter (Crampton, 1977; Kokelj et al., 2007). However, whether recent hummock development and tree leaning are accelerated by climate warming or occur as natural phenomena remains unclear.

To analyze the effects of climate change on drunken forest soil hummock dynamics, we focused on a unique trait of conifers, the formation of reaction wood. Leaning conifer trees reinforce their trunks by forming wider, darker tree rings (Stoffel & Bollschweiler, 2008; Yamashita et al., 2009). Thus, tree leaning events caused by mound slope steepening are recorded in reaction wood disks (Stoffel & Bollschweiler, 2008). Dendrochronological analyses allow us to reconstruct the history of disturbance events, as well as past climate.

We selected open black spruce woodlands in the Mackenzie upland area near Inuvik, northeastern Canada, as a natural laboratory for climate warming; this region is a climate warming hotspot, with a greater increase in mean annual air temperature (+3.5°C, 1961–2010) than the global average (+0.5°C to +0.7°C per 100 years; Akasofu, 2010; IPCC, 2021). Increases in plant productivity and active layer thickness were observed under warmer climate in some locations near Inuvik (Mackay, 1995; Tei et al., 2017). Up to 200 years of environmental change are recorded in the tree rings of this region (Tei et al., 2017; Zoltai, 1975). Different geological substrates including glaciofluvial sands and fine‐grained clayey sediments create widely variable permafrost table depths and mound sizes (Figure 1d). Tree ring records in reaction wood allow us to reconstruct soil hummock and drunken forest development before and after warming.

**FIGURE 2:**
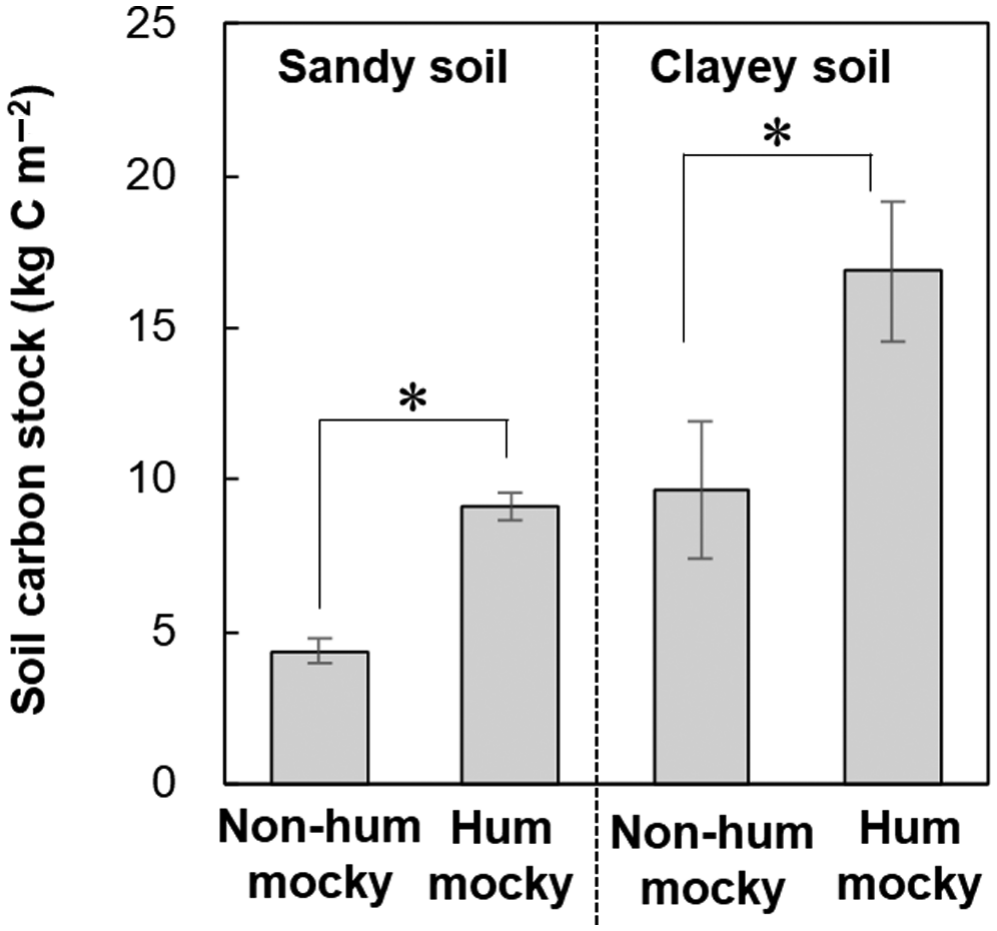
Soil carbon stocks in hummocky and non‐hummocky microrelief areas with sandy and clayey sediments. Carbon stocks in the organic horizon and mineral soil (0–30 cm depth) were measured by multiplying the soil carbon concentration × bulk density × individual depth (Table S1). Bars indicate standard error (*N* = 6, 6, 3, and 3 for hummocky clayey soil, non‐hummocky clayey soil, hummocky sandy soil, and non‐hummocky sandy soil, respectively). Asterisks indicate significant differences between groups evaluated using analysis of variance (ANOVA; *p < .05), n.s., non‐significance. Data source: Table S1

Because black spruce forest soils with hummocky microrelief exhibit greater carbon storage potential than non‐hummocky soils (Fujii et al., 2019; Figure 2; Table S1), the responses of drunken forests and hummocks to climate change may affect soil carbon dynamics on warming permafrost (Schuur et al., 2015). In this study, we reconstructed the development of drunken forest and hummocks before and after warming to test whether contemporary drunken forest and hummock development is affected by recent warming.

## 2 MATERIALS AND METHODS

### 2.1 Experimental design

To reconstruct the development of soil hummocks and drunken trees, we analyzed black spruce (*Picea mariana* L.) tree ring records and soil microrelief in the Northwest Territories, Canada (N68°03′, W133°30′). We collected a total of 50 tree disks from 50 hummocks between Inuvik and Tsiigehtchic (Tables S1 and S2). This region has a subarctic climate, with a mean annual air temperature of −8.8°C. Annual precipitation was 248 mm year^−1^, of which snow amounted to 131 mm year^−1^. The soil is underlain by continuous permafrost that developed during ice ages without coverage by the Laurentide ice sheet (Dyke, 2004).

We compared two soil types (clayey and sandy soils) with contrasting permafrost table depths and mound sizes (Table S2). Clayey soils are derived from a mixture of fluvial clayey sediments and fine‐grained glacial till (27%–37% clay and 22%–30% sand), whereas sandy soils are derived from glaciofluvial sands (8%–12% clay and 73%–75% sand). To analyze changes in mound growth and tree leaning before and after warming, we compared young trees born after 1910 and mature trees born before 1860 (>50 and <100 years old in 1961, respectively).

### 2.2 Soil and microrelief survey

We measured the hummocky microrelief of the soil surface (relative elevation) and maximum depths from the thawed soil to the permafrost table in August (active layer thickness) on the mound and depression sides surrounding tree stumps, at a distance of 20 cm from the trunk. Hummocky microrelief showed differences in active layer thickness between the mound and depression sides surrounding tree stumps. The ratios of the maximum and minimum active layer depths to the permafrost table were used as an indicator of hummock size (Table S2).

### 2.3 Tree disk sampling and dendrochronological analysis

Tree disk samples were collected by cutting the tree stems perpendicular to the stem axis at a height of 30 cm, where reaction wood formation reaches its maximum (Fujii et al., 2020; Figure 4a,b). Wood tissues were observed through optical microscopy after staining the lignin and non‐lignin tissues (protein and cellulose) using safranin and fast green dyes, respectively (Figure 4c,d). We applied dendrochronological analysis to estimate disturbance events (i.e., soil hummock formation). Tree ring annual widths were measured using a stereomicroscope (MZ6; Leica) and tree ring measurement system (Velmex Inc.) in two directions along the major axis of the wood disk, including the maximum wood radius, and two perpendicular directions on the minor axis, for a total of four radius measurements. To assess the validity of tree ring records with high density (e.g., 10 tree rings per mm), tree ring data were visually cross‐dated and statistically analyzed using the COFECHA program (Holmes, 1983); the results are shown in Fig. S1.

### 2.4 Detection and reconstruction of tree leaning and hummock formation events

The annual intensity of tree leaning was defined as the ratio of maximum annual tree ring width to average annual tree ring width (Data S1) and calculated by dividing the maximum annual tree ring width by the average annual ring width in the other three directions (±90° and 180° relative to the maximum tree ring width). Peaks of annual intensity of tree leaning >2 were identified as active tree leaning events, during which the trees produced round cells (reaction wood); the percentage of active tree leaning events is shown in Figure 6c. After this filtering step, peaks in the annual intensity of tree leaning exceeding those of the previous year were counted as new mound rising events (Figure 6c).

To reconstruct hummock development, tree ring widths were summed to calculate wood increment curves (Data S2). Then, the extent of reaction wood formation was calculated from the ratio of maximum wood radius to average wood radius (Figure 1e). Reaction wood formation >1 indicated that trees were leaning toward the orientation of the mound slope.

### 2.5 Statistical analyses

Mean reaction wood formation and annual intensity of tree leaning were calculated. Meteorological data were obtained from the nearest meteorological station to the study site (Inuvik, 1958–2014), and missing data (1911–1958, 1971) were estimated using data from the nearest station (Fort Good Hope, 1911–2014). Arctic annual air temperature anomalies (1880–2018) were obtained from NASA Goddard Institute for Space Studies and the missing data (1820–1879) were estimated using data of arctic‐wide summer air temperature anomalies inferred from the existing tree ring record (Jacoby & D’Arrigo, 1989). To compare formation of reaction wood before and after warming, the extents of reaction wood formation were plotted against tree age (up to 150 years old) and differences in linear regression slopes between young and mature trees were tested analysis of covariance (ANCOVA). To compare tree leaning activity before and after warming, tree leaning activities (ln‐transformed) were plotted against tree age and differences in regression slopes between young and mature trees were tested using ANCOVA. All statistical tests were performed using the SigmaPlot software v14.0 (SPSS Inc.).

## 3 RESULTS

### 3.1 Soil microrelief and tree ring observations

**FIGURE 3:**
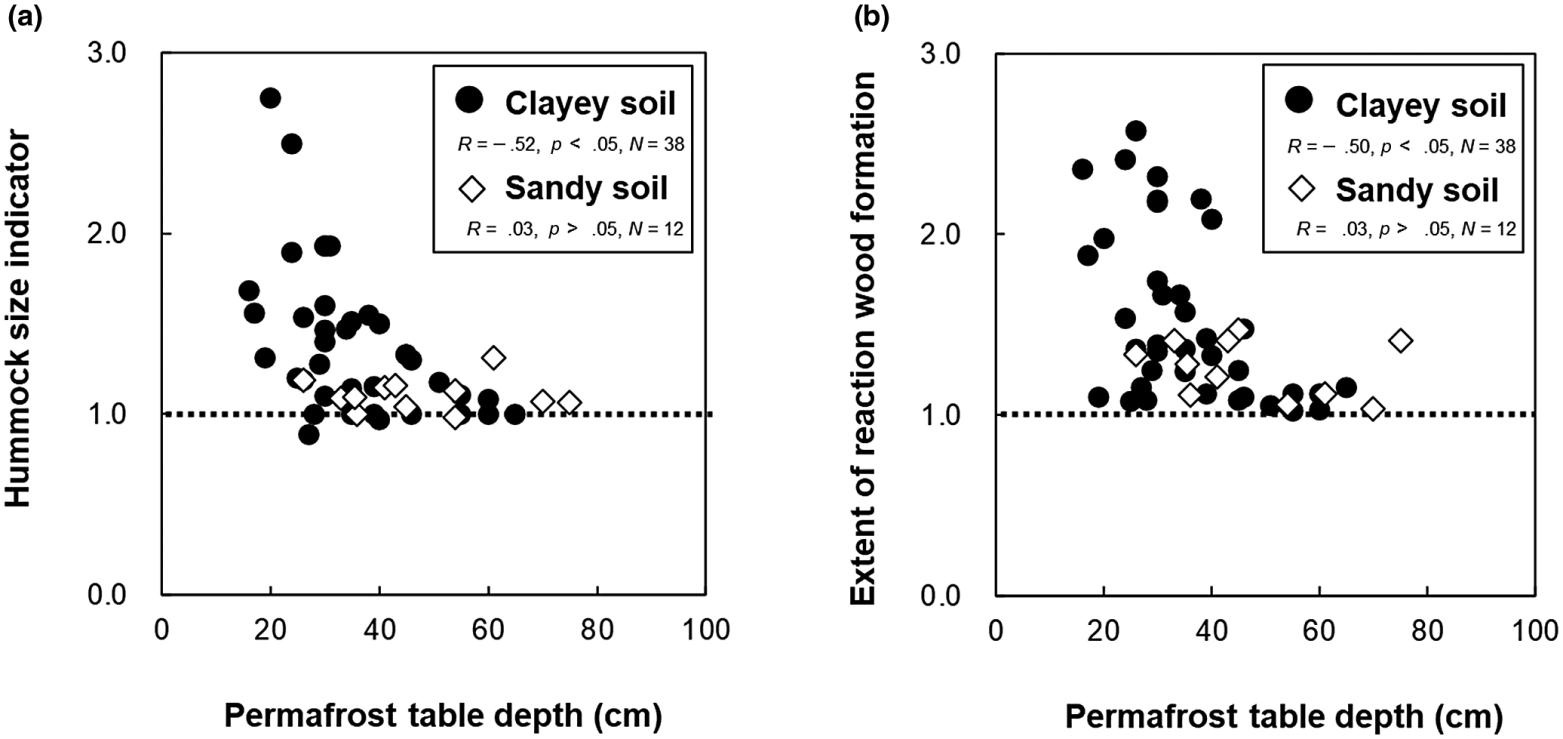
Relationships between hummock development, reaction wood formation, and permafrost table depth. (a) Hummock size indicator (Table S2) related to permafrost table depth in clayey and sandy soils. (b) Extent of reaction wood formation (Table S2) related to permafrost table depth in clayey and sandy soils. Both hummock size and tree leaning decreased as permafrost table or active soil layer depth increased in clayey soils. Data source: Table S2

To test whether drunken forest development is related to hummock formation, we analyzed the relationship between mound size and reaction wood formation (ratio of maximum to average wood radius). Trees standing straight on flat microrelief, including mound tops, produce regular circular tree rings, whereas most trees growing on mound edges have wider tree rings (eccentric growth) at the downslope side of the mound than on the mound top (Figure 1d). The hummock size indicator (ratio of maximum to minimum active layer depth) was positively correlated with reaction wood formation (Figure 1d). Both maximum and average tree ring width decreased gradually with tree age among normal disks, whereas maximum and average tree ring width differed greatly in some years in reaction wood (Figure 1e). The cumulative effects of these events led to reaction wood formation (Figure 1e). Changes in the extent of reaction wood formation indicated that trees gradually lean toward the downslope side as the mound slope steepens (Figure 1e). There were negative correlations between permafrost table depth and hummock size indicator (Figure 3a) and between permafrost table depth and extent of reaction wood formation (Figure 3b) for the clayey soils, but not for the sandy soils.

**FIGURE 4:**
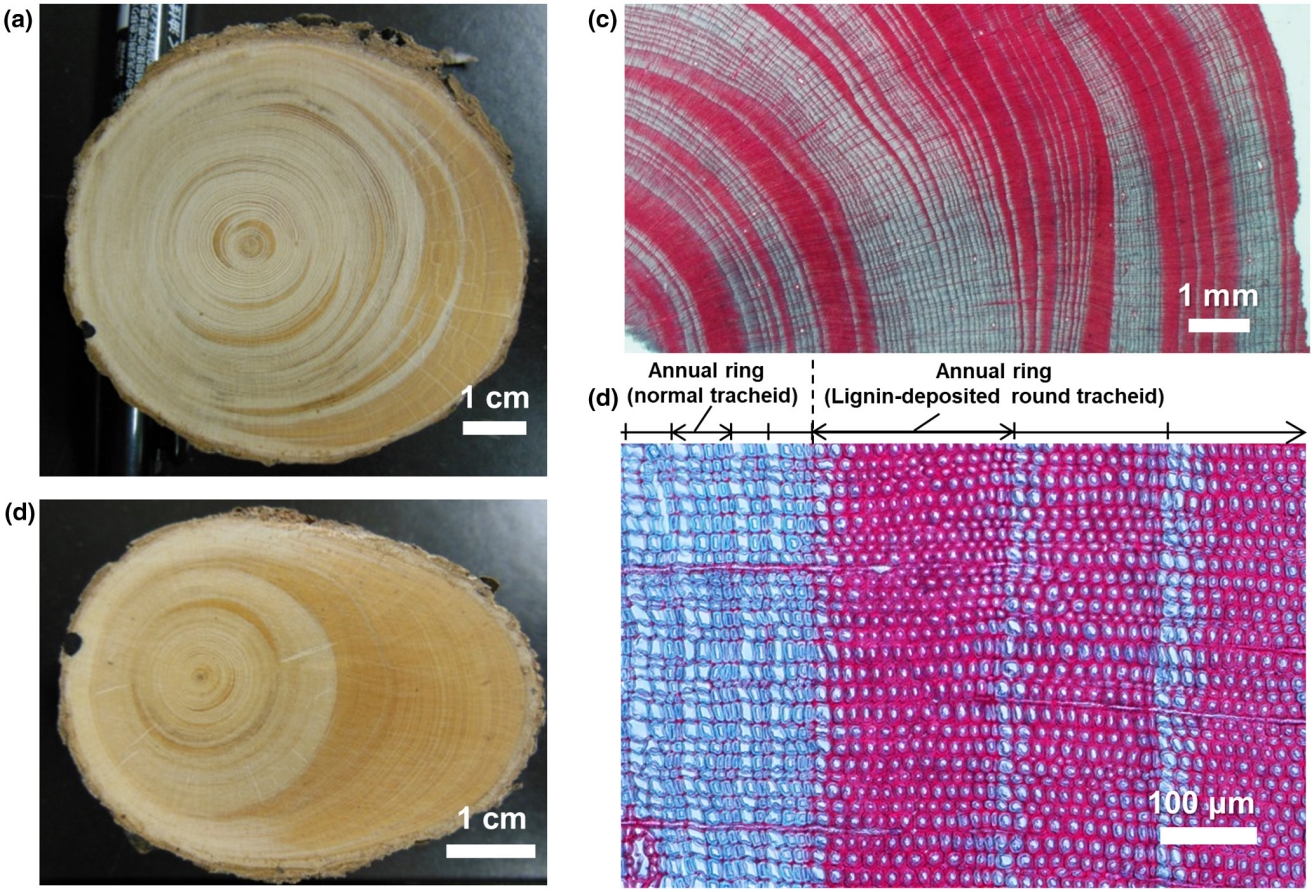
Cross‐sections of leaning trees. (a, b) Wood disks recording tree leaning events (reaction wood). (c, d) Tree rings containing round, lignin‐rich cells (red cells after double‐staining with safranin and fast green dyes) observed through optical microscopy. Bars: 1 cm (a, b), 1 mm (c), and 100 μm (d)

To analyze the timing and driving mechanism of tree leaning, we observed when lignin deposition started in the annual tree ring cells (Figure 4a,b). The lignin‐rich round cells in the reaction woods were observed in the earlywood or the initial stage of annual ring production (Figure 4c,d). The gradual increase in lignin‐rich round cells in latewood was minor (<10%) in our study.

### 3.2 Reconstruction of soil hummock formation and drunken forest development

**FIGURE 5:**
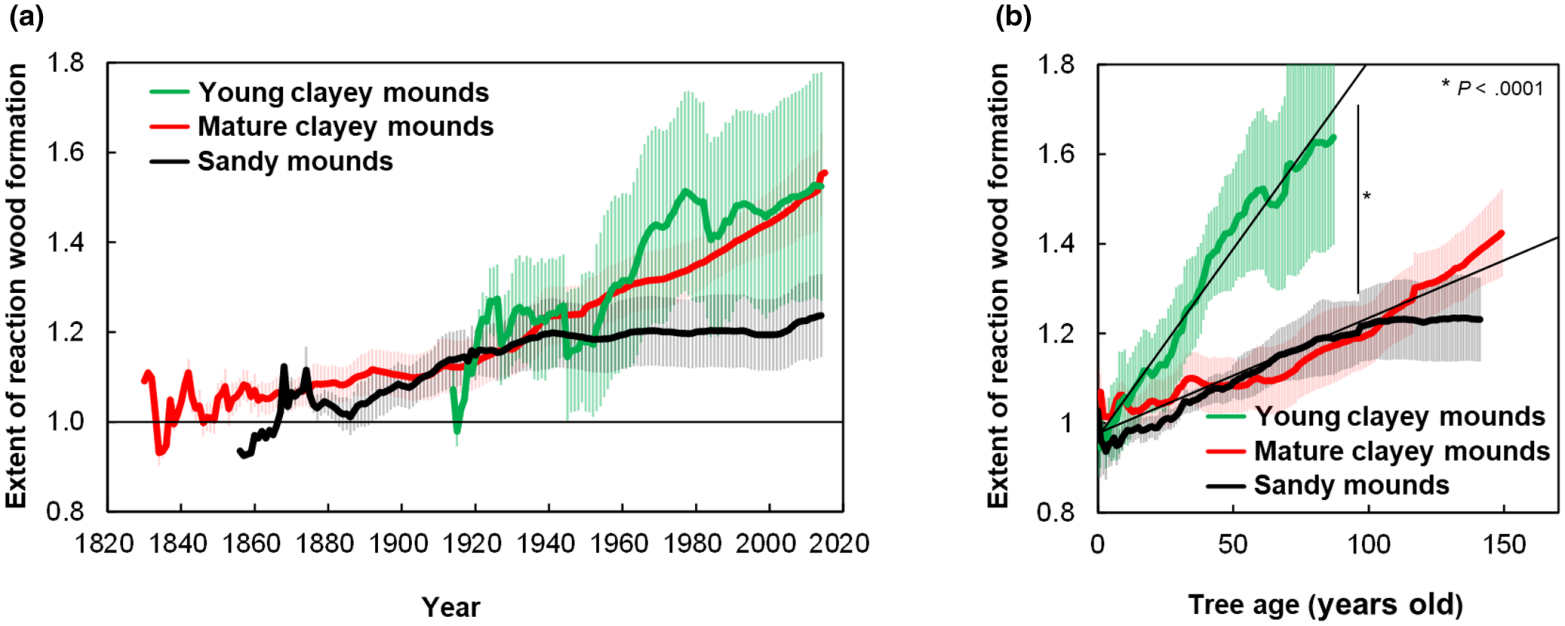
Soil hummock and drunken forest development. (a) Time series trends of reaction wood formation extent, recording soil hummock development. (b) Relationships between mound age (tree age) and reaction wood formation. Error bars in Figure 3a,b indicate standard errors (*N* = 6, 20, and 6 for young clayey mounds, mature clayey mounds, and sandy mounds, respectively)

Among 50 tree disks examined in this study, 28 satisfied our dendrochronological quality check (cross‐dating by the COFECHA program and microscopic observation); therefore, we successfully reconstructed the development of 28 soil mounds, with 22 clayey mounds having formed on fine‐grained sediments and six sandy mounds on glaciofluvial sands (Figure 5a). Clayey mounds continued to grow over a 200‐year period, whereas sandy soil growth reached saturation within 70 years (Figure 5a). When the growth curves of clayey mounds with young and mature trees were compared for the same tree ages, the growth rates of young mounds under a recently warmer climate was significantly higher than that of mature mounds developed under past cooler climates (Figure 5b).

**FIGURE 6:**
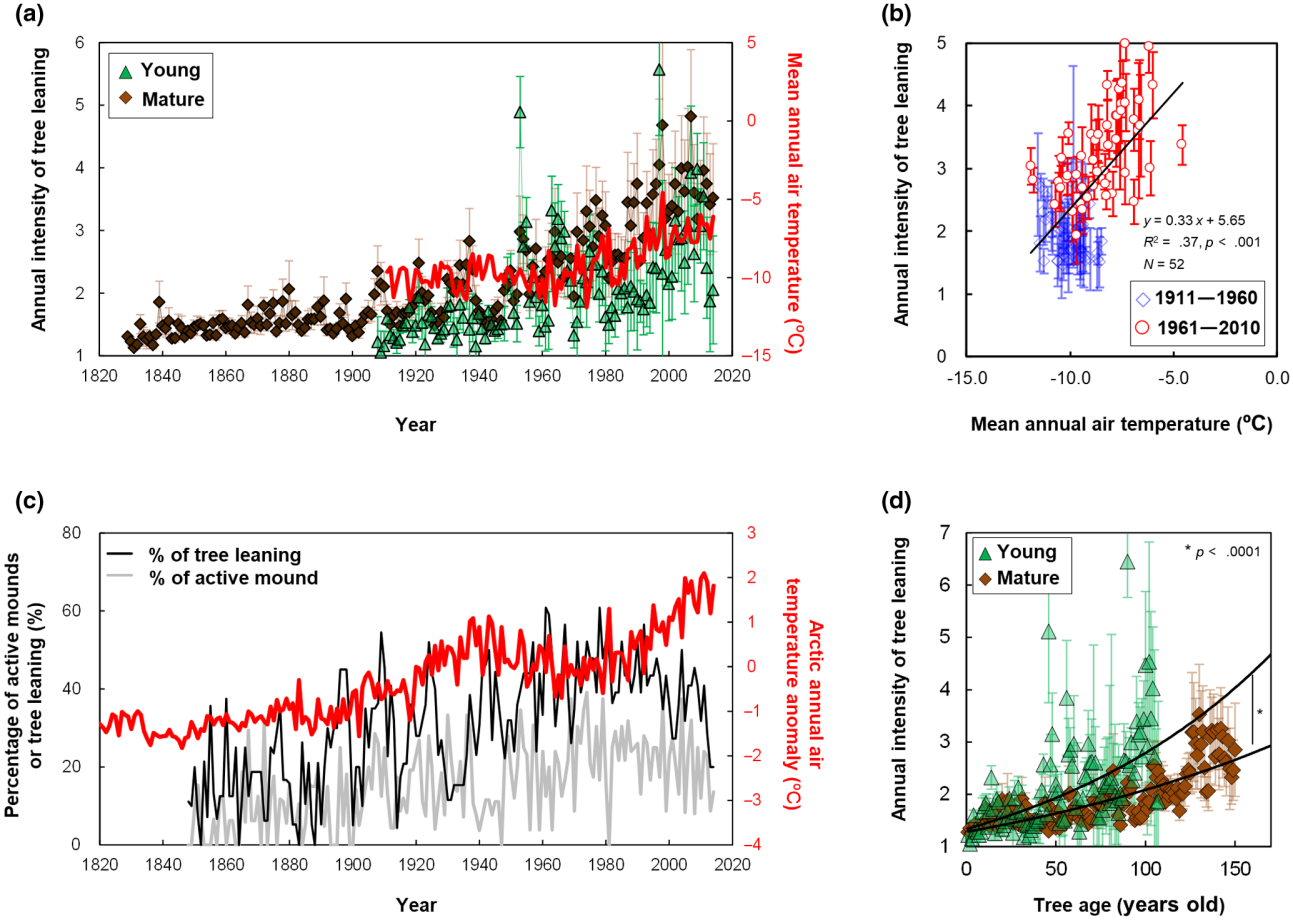
Annual activities of tree leaning. (a) Time series trends of tree leaning and climate warming. (b) Correlation between annual intensity of tree leaning and air temperature. (c) Time series trends of arctic annual air temperature and percentages of active clayey mounds and tree leaning. (d) Relationships between annual intensity of tree leaning and tree age (0–100 years old). Error bars in a, d indicate standard errors (*N* = 6 and 20 for young and mature trees, respectively), whereas those in (b) indicate standard errors (*N* = 26)

We compared the annual intensity of tree leaning and hummock formation before (1850–1961) and after warming (1961–2010) and found that the annual intensity of tree leaning had increased since the advent of recent warming (Figure 6a). We detected a positive correlation between annual tree leaning intensity and the air temperature of the previous year during 1961–2015 (Figure 6b). The magnitude and frequency of hummock development also changed after the advent of recent warming (Figure 6c). The percentages of active clayey mounds and tree leaning were positively correlated with the arctic annual air temperature of the previous year, respectively (*R* = 0.4–0.5, *N* = 167, *p* < .01; Figure 6c). The interval between active mound‐rising events was shorter after warming (3.0 years) than the interval before warming (5.9 years; Figure 6c). When we compared the annual intensity of tree leaning at the same age between the mature trees growing before warming (1850–1961) and young trees after warming (1961–2010), young trees under recent warming had the higher leaning intensity, compared to mature trees that had experienced the young age before warming (Figure 6d).

## 4 DISCUSSION

### 4.1 Relationship between soil hummock formation and drunken forest development

We detected a positive correlation between hummock growth and reaction wood formation (Figure 1d), supporting the hypotheses that hummock formation is the main driver of drunken forest development in continuous permafrost areas and that reaction wood tree rings record drunken forest and soil hummock development (Figure 1e). This finding is consistent with the distribution of drunken forests and hummocky ground; hummocky microrelief increases from the southern discontinuous permafrost zone (<5% of terrain at Fort Simpson) to the northern continuous permafrost zone, which contains drunken forests (80% of terrain in Inuvik; Tarnocai & Zoltai, 1978). In the continuous permafrost zone, drunken forests develop along with soil hummock formation (Figure 1d,e), which is in contrast to drunken forest phenomena around thermokarst formation through permafrost degradation in the discontinuous permafrost zone (Schuur & Abbott, 2011).

### 4.2 Timing of drunken forest development on continuous permafrost

Reaction wood records disturbance and soil hummock formation events (Crampton, 1977). Active tree leaning events always produce lignin‐rich round cells with thick cell walls (Figure 4c,d) because lignin production is immediately triggered by gravitational imbalance in leaning trees (Yamashita et al., 2009). Tree leaning induced by permafrost degradation in warming summers should cause a gradual shift from normal tree rings to lignin‐rich round cells in annual rings. However, we found that formation of lignin‐rich round cells began in the early growing season (earlywood), not the later growing season (latewood; Figure 4c,d). The abrupt transition from normal tree rings to reaction wood suggests the occurrence of tree leaning events during winter (Figure 4). Because tree leaning is synchronized with hummock formation (Figure 1d,e), tree leaning is caused by mound rising in winter. This conclusion was supported by a recent survey, which reported that most tree leaning followed the direction of the mound slope, rather than wind or snow effects that force trees to tip in various directions (Fujii et al., 2020). In drunken forests on discontinuous permafrost, permafrost degradation induces soil foundation loosening and tree leaning in warming summers (Schuur & Abbott, 2011), whereas hummock formation by winter soil freezing was the main driver of drunken forest development on continuous permafrost in our study.

### 4.3 Climate warming accelerates soil hummock and drunken forest development

Polygon formation occurs at geological time scales (Tarnocai & Zoltai, 1978), whereas drunken forest development occurs with hummock formation in forest fire regeneration cycles (Figure 1d,e). During 1981–1970, mound growth reached saturation within 150 years (Zoltai, 1975), whereas clayey mounds continued to grow for approximately 200 years in our study (Figure 5a). Soil hummock development occurred occasionally before warming (1911–1960) but shifted toward continuous mound rising and tree leaning (Figure 6c,d). These differences may be attributable to recent climate warming because soil hummock formation activity increased with air temperature (Figure 6b). An increase in reaction wood percentage was also observed in Yukon (1960–1970; Huisman, 2002). Active mound growth requires a warm climate as a driver of soil movement (Bockheim, 2007) in addition to the preconditions of a shallow permafrost table, high ice content, and the structural stability of clayey soils (Fujii et al., 2019). The refreezing of deeply thawed soil generates greater heaving pressure in warmer years than in colder years (Figure 6b). Active mound rising leads to continuous tree leaning unless trees regain vertical orientation prior to the subsequent event (Figure 6c). Development of drunken forest was also reported in the discontinuous permafrost zone of Siberia, where an increase in summer precipitation, rather than warming, induced the thermokarst formation and tree leaning (Agafonov et al., 2004). Although both global climate change and site‐specific parameter (slope and tree age) affects tree leaning, an intense warming (+3.5°C, 1961–2010) and clayey soil texture might be favorable conditions to show direct evidence of rapid drunken forest development in our study.

Because black spruce trees have no distinct taproot, they expand their main root system towards deeper and warmer mounds, escaping depressions on the shallow permafrost table (Fujii et al., 2020). We speculate that mature trees, having greater weight and root expansion, promote tree leaning toward the mound slope under warming‐induced mound rising. However, the higher growth rates of recent clayey mounds observed in this study suggest that drunken forest development depends on warmer climate, rather than tree age (Figure 5b). Similarly, leaning activity was found to be greater for young trees under a recent warm climate than that of mature trees that experienced the past colder climate at a similar age (Figure 6d). Therefore, warming‐induced soil movement, rather than tree age, appears to be the major cause of recent tree leaning.

### 4.4 Potential impacts of climate warming on drunken forests

We found dendrochronological evidence that climate change accelerated the development of drunken forests and soil hummocks (Figure 6c). Soil hummocky microrelief provides lichen and moss habitats, which produce recalcitrant litter (Lang et al., 2009; Turetsky et al., 2011) and display greater carbon sequestration in soil compared to non‐hummocky soil covered by vascular plants (Figure 2). On the other hand, warming increases fire susceptibility and affects soil carbon cycles (O'Donnell et al., 2011). The development of dry lichen‐covered mounds might increase fire susceptibility and risk shortening fire‐regeneration cycles of drunken forest and residence time of ecosystem carbon stored. In addition, a shallow permafrost table is the precondition for continuous soil hummock development (Figure 3a,b; Fujii et al., 2020). Deepening of the permafrost table or permafrost degradation by further warming is unfavorable for soil hummock development and carbon storage (Figures 2 and 3a,b; Plaza et al., 2019). Thus, although soil changes are generally slow, tree ring records suggest that recent warming threatens the stability of drunken forests and organic carbon in soil hummocks.

## 5 CONCLUSION

Although drunken forest phenomena have been widely regarded as a symptom of permafrost degradation in warming summer in the discontinuous permafrost zone, drunken forests develop along with soil hummock formation in the continuous permafrost zone. We found that tree leaning is caused by mound rising in winter due to refreezing of soil following deep thaws in summer, rather than by loosening of the soil foundation in summer. On the other hand, hummock formation shifted from periodic events before 1960 to continuous mound rising in the warmer succeeding 50 years. We found dendrochronological evidence that tree leaning and soil hummock formation have accelerated due to recent climate warming. Judging from development of drunken forests and soil hummocks requires the presence of shallow permafrost table, recent warming threatens the stability of drunken forests and organic carbon in soil hummocks.

## CONFLICT OF INTERESTS

None declared.

## AUTHORS’ CONTRIBUTIONS

K.F. designed the study and wrote the manuscript. K.Y. performed dendrochronological measurements. Y.M. established the field for soil survey.

## Supporting information

Fig S1

Table S1

Table S2

## Data Availability

The data that support the findings of this study are openly available in https://datadryad.org/stash/dataset/doi:10.5061/dryad.dr7sqv9z5.
