# Supplementary figures and images for: Tree ring evidence of rapid development of drunken forest induced by permafrost warming
Source: Glob Chang Biol. 2022 Apr 7;28(12):3920–8. doi: 10.1111/gcb.16176 (PMC9324869; doi:10.1111/gcb.16176)

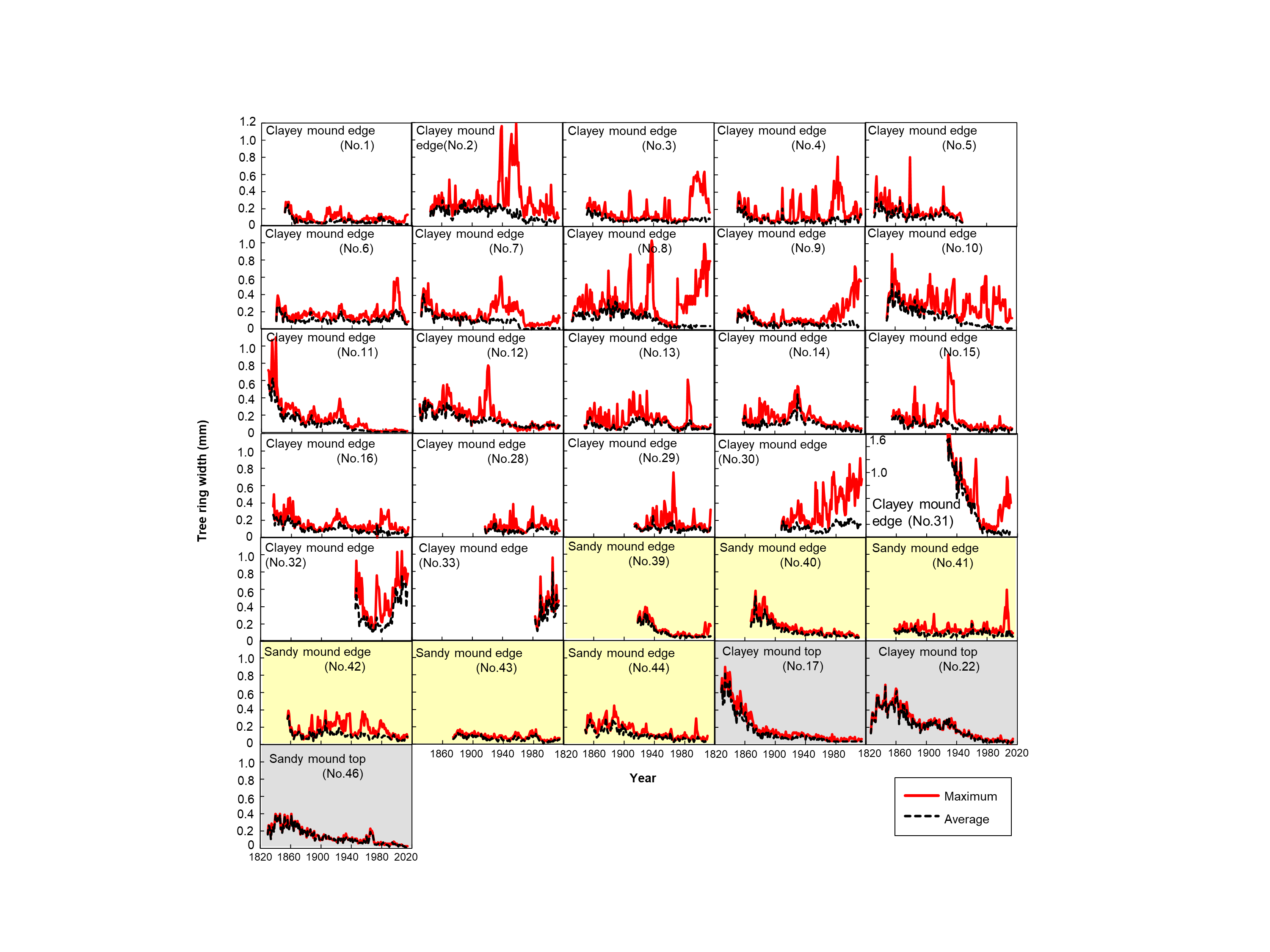

Supplement: Supplementary file 1 — Fig S1 [file GCB-28-3920-s002.tif]

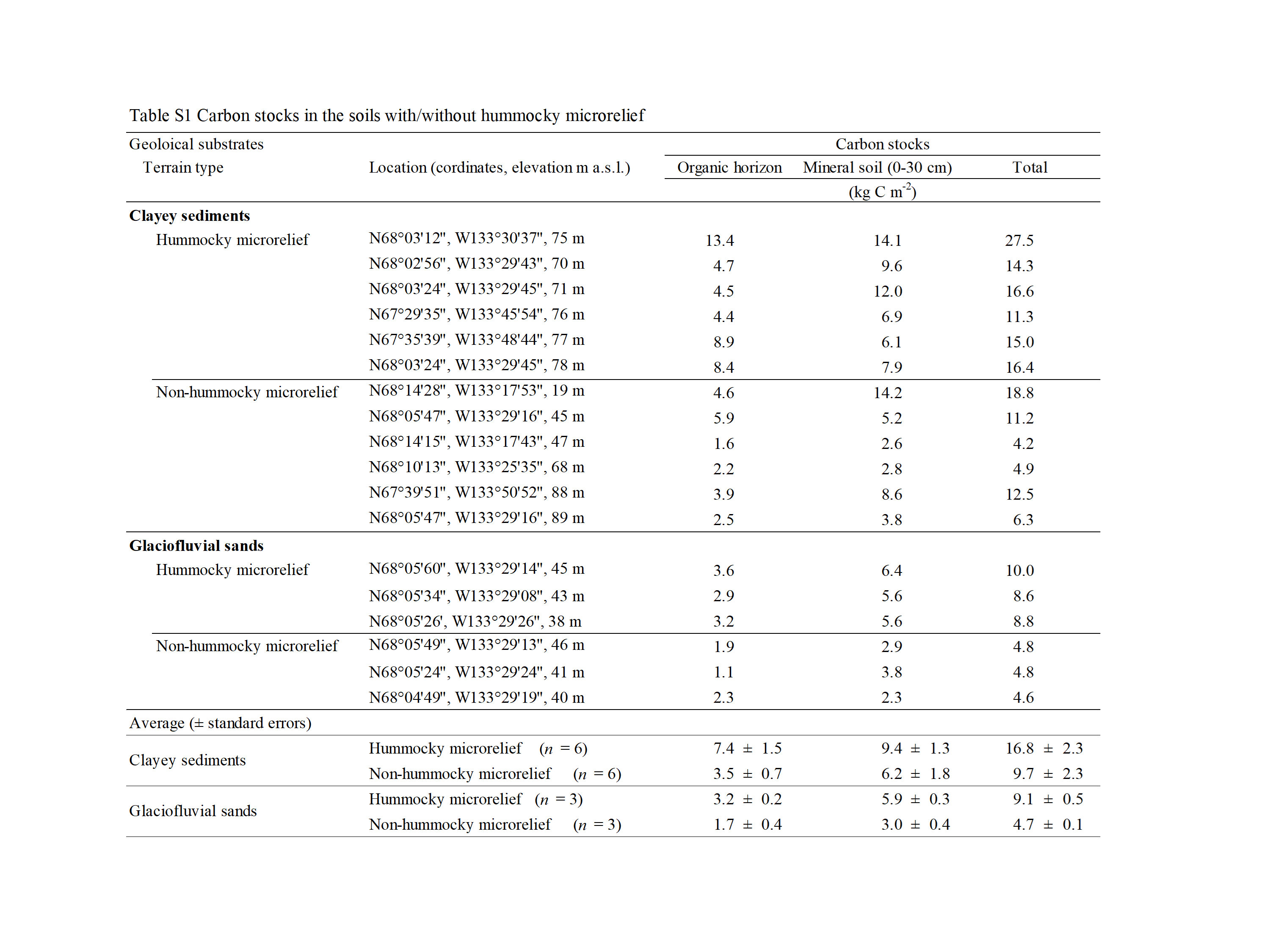

Supplement: Supplementary file 2 — Table S1 [file GCB-28-3920-s001.tif]

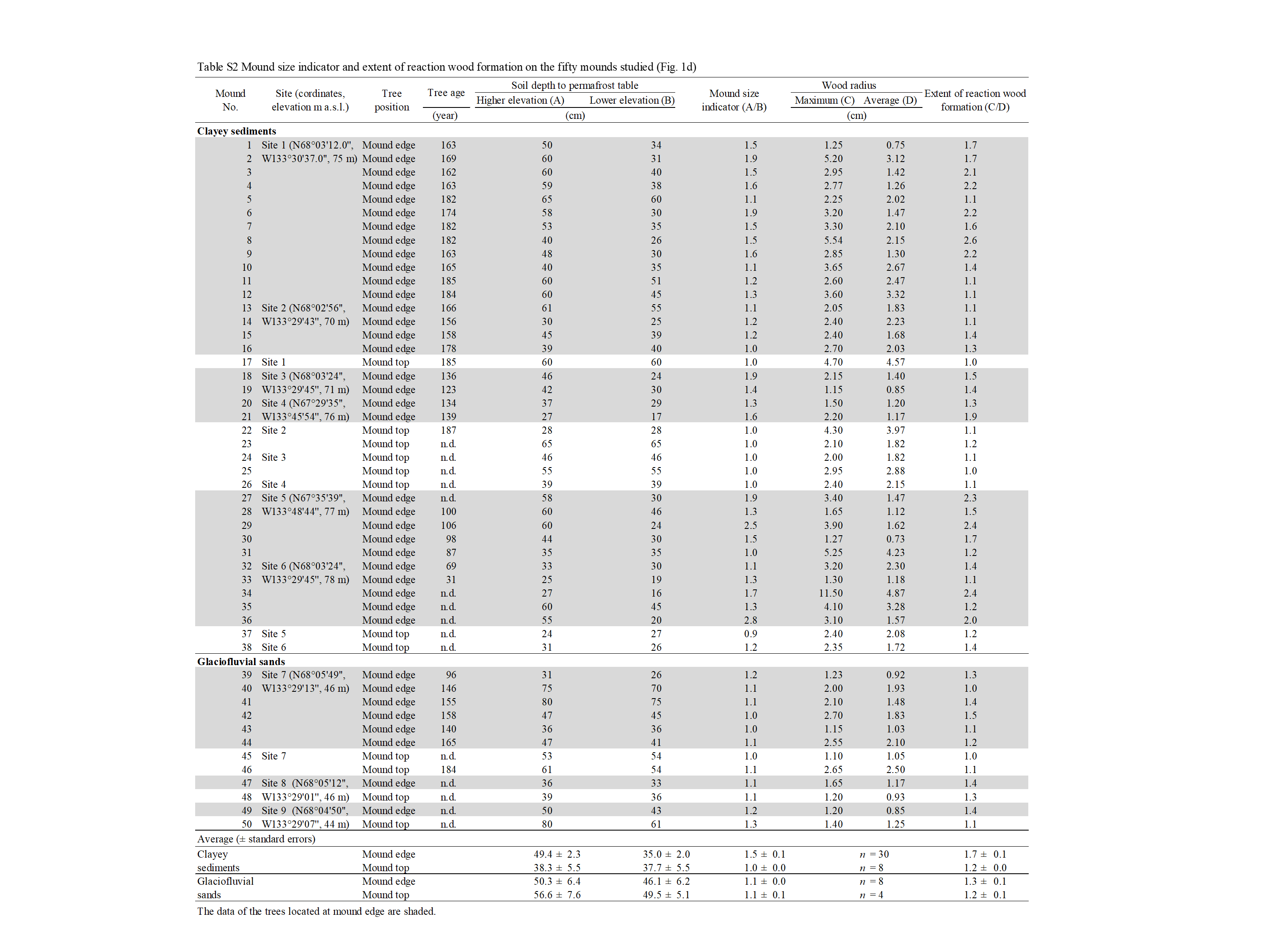

Supplement: Supplementary file 3 — Table S2 [file GCB-28-3920-s003.tif]
